# TGIF2 promotes the progression of lung adenocarcinoma by bridging EGFR/RAS/ERK signaling to cancer cell stemness

**DOI:** 10.1038/s41392-019-0098-x

**Published:** 2019-12-13

**Authors:** Renle Du, Wenzhi Shen, Yi Liu, Wenjuan Gao, Wei Zhou, Jun Li, Shuangtao Zhao, Chong Chen, Yanan Chen, Yanhua Liu, Peiqing Sun, Rong Xiang, Yi Shi, Yunping Luo

**Affiliations:** 1 Department of Immunology, School of Medicine, Nankai University, Tianjin, 300071 China; 2 Department of Pathology and Institute of Precision Medicine, Jining Medical University, Jining, 272067 China; 3 Department of Immunology, Institute of Basic Medical Science, Chinese Academy of Medical Science, School of Basic Medicine, Peking Union Medical College, Beijing, 100005 China; 4 2011 Project Collaborative Innovation Center for Biotherapy of Ministry of Education, Tianjin, 300071 China; 5 Tianjin Key Laboratory of Tumour Microenvironment and Neurovascular Regulation, Tianjin, 300071 China; 6 Department of Cancer Biology, School of Medicine, Wake Forest University, Winston-Salem, NC 27157 USA

**Keywords:** Cancer stem cells, Metastasis, Lung cancer, Oncogenes

## Abstract

TGF-β-induced factor homeobox 2 (TGIF2) is a transcription regulator that plays essential roles in the regulation of development and cell fate decisions. Aberrant expression of TGIF family proteins has been observed in several cancers, including ovarian, esophageal, and colorectal cancers. Here, we report that TGIF2 mediates the EGFR–RAS–ERK signaling pathway to enhance the stemness of lung adenocarcinoma (LUAD) cells and, therefore, promote the progression and metastasis of LUAD. We found that high TGIF2 expression was closely correlated with tumor growth, lymph node metastasis, and survival of patients with LUAD. Mice bearing TGIF2-silenced H1299 xenografts developed smaller tumors and fewer lung metastases. Importantly, silencing TGIF2 decreased the cancer stem cell (CSC)-like properties in A549 and H1299 cells. Furthermore, we identified that TGIF2 binding to the *OCT4* promoter promotes its expression. In both LUAD cells and in vivo LUAD mouse models, we revealed that EGFR–RAS–ERK signaling phosphorylated TGIF2 and increased its stability, which was important for TGIF2-promoted LUAD stemness since phosphorylation-deficient TGIF2 mutants lost these functions. Thus, our study revealed that an important factor, TGIF2, bridges EGFR signaling to the CSC characteristics of LUAD cells, which can be utilized as an effective target for LUAD therapy.

## Introduction

Lung cancer is the leading cause of cancer-related death worldwide, and non-small cell lung cancer (NSCLC) accounts for approximately 85% of all types of lung cancers.^1^ NSCLC is typically diagnosed at advanced stages, resulting in a poor 5-year survival rate of less than 15%.^2^ Intratumoral heterogeneity is considered one of the major reasons for the limited efficacy of current anticancer therapies.^3^ It has been well documented that cancer stem cells (CSCs), a subpopulation of neoplastic cells capable of initiating new tumors,^4,5^ are the main cause of intratumoral heterogeneity.^6^ Of note, the CSC phenotype is intimately interconnected with activation of the epithelial**–**mesenchymal transition (EMT) program,^7–9^ which is an important step during tumor metastasis. Although the mechanisms of CSC maintenance have been intensively studied, the key genes that regulate CSCs in NSCLC, especially caused by aberrant EGFR activation, remain elusive.

TGF-β-induced factor homeobox 2 (TGIF2) is a member of the three-amino acid loop extension (TALE) superfamily of homeodomain proteins that play important roles in the regulation of many crucial developmental programs, including cell proliferation and differentiation. TGIF2 acts as a transcriptional repressor either by interacting with the TGF-β/BMP pathways or by binding to DNA directly.^10,11^ Recently, TGIF2 has been reported to be able to enhance gene expression. In melanoma, TGIF2 directly upregulates the transcription of *FUT8* (Fucosyltransferase 8) to induce metastasis, leading to melanoma aggressive behavior.^12^ Moreover, TGIF2 could bind to the *CDH1* promoter and activate CDH1 expression in the epithelial state of colon cancer cells.^13^ In addition, TGIF2 was recently reported to be a key developmental regulator of the stepwise reprogramming of liver cells to a pancreas progenitor state.^14^ During this progression, forced expression of TGIF2 was able to give rise to a higher number of upregulated than downregulated pancreatic progenitor genes, suggesting that TGIF2 may simultaneously act as both a transcriptional activator and a repressor. These characteristics of TGIF2-mediated transcriptional regulation are consistent with other TALE homeoproteins showing context-dependent activities. TGIF2 proteins have been reported to be upregulated in several cancer types including ovarian and colorectal cancers.^15,16^ However, the role of TGIF2 in NSCLC remains largely unexplored.

Epidermal growth factor (EGF) plays an important role in regulating cell growth, proliferation, and differentiation. It has also been implicated in cancer stemness and EMT.^17,18^ EGF stimulates multiple biological responses through activation of the EGF receptor (EGFR), and activated EGFR phosphorylates and activates a number of important signaling pathways.^19^ RAS/RAF/MAPK is considered one of the traditional downstream effectors of EGF/EGFR. EGFR/RAS/ERK signaling is often aberrantly activated in cancer, resulting in cell proliferation, malignant transformation, and drug resistance.^20–22^ Furthermore, this pathway can directly phosphorylate numerous transcription factors, including ETS-1, c-JUN, and c-MYC. TGIF2 has been reported to be phosphorylated by EGF/RAS/ERK signaling.^8^ However, the function of TGIF2 triggered by this pathway is still unclear.

In the present study, we investigated the function and mechanism of TGIF2 in promoting the progression of lung adenocarcinoma (LUAD) in vitro and in vivo. We demonstrated that TGIF2 phosphorylation induced by EGFR/RAS/ERK signaling promotes OCT4 expression, leading to increased stemness and metastasis of LUAD cells. The identification of TGIF2 as a key regulator bridging EGFR signaling to the stemness of LUAD cells provided novel insights into EGFR-induced metastasis and drug resistance of LUAD, indicating that TGIF2 could be a potential therapeutic target for LUAD.

## Results

### High expression of TGIF2 correlates with the poor prognosis of patients with LUAD

**Fig. 1:**
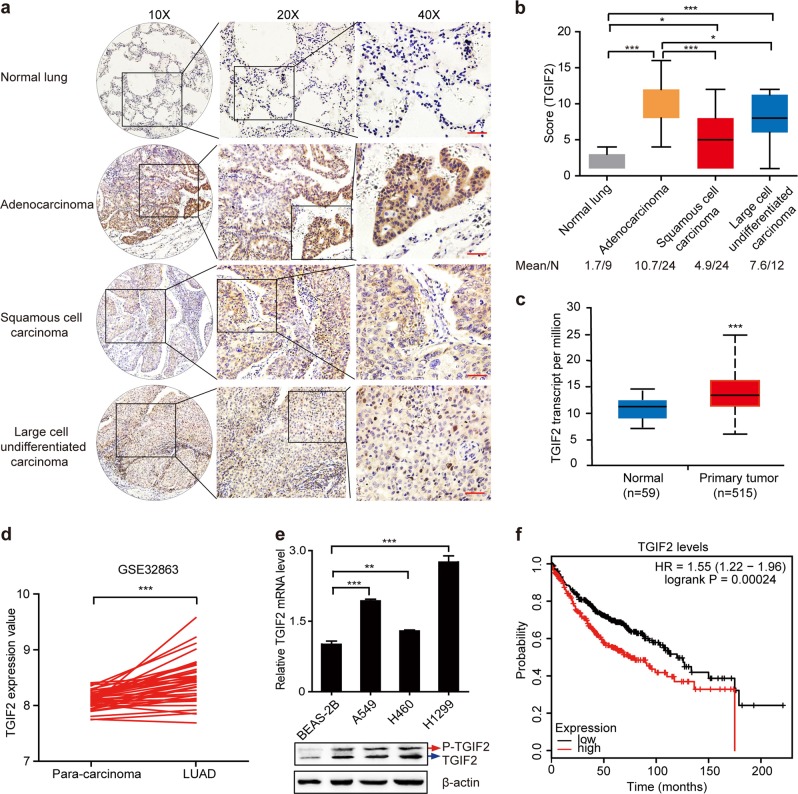
Upregulation of TGIF2 in LUADs and its association with prognosis. **a** Immunohistochemical analysis of TGIF2 protein expression using human NSCLC tissue microarrays (including 24 lung adenocarcinoma, 24 squamous cell carcinoma, 12 large-cell undifferentiated carcinoma, and 9 normal lung samples, each with three replicates). Scale bars: 50 μm. **b** Analysis of TGIF2 expression in the different NSCLC tissues according to staining score. The number of samples and the mean values of each group are listed below the *x*-axis. **c** Transcript levels of TGIF2 in 59 normal tissues and 515 LUAD primary tumors from TCGA (*P* < 0.001 [unpaired *t*-test]). **d** *TGIF2* mRNA expression in LUAD compared with matched adjacent normal lung tissues in GSE32863 (*n* = 36; *P* < 0.001 [paired *t*-test]). **e** TGIF2 expression in a normal lung cell line (BEAS-2B) compared with NSCLC cell lines (A549, H1299, H460) as examined by qRT-PCR (bar plot, upper panel) and western blotting (lower panel). Data are shown as means ± SD. **P < 0.01, ***P < 0.001. **f** Kaplan–Meier curves showing the survival of 2437 patients with LUAD with different *TGIF2* gene expression levels ([log-rank test], HR, hazard ratio).

Elevated TGIF2 levels have been reported in ovarian cancer and colorectal carcinoma.^15,16^ Yadong Wang et al. also reported high expression of TGIF in lung carcinogenesis using a cell-based in vitro system.^23^ To explore the real correlation between TGIF2 levels and LUAD progression in human patients, we first examined the TGIF2 protein levels of 60 human NSCLC specimens and 9 normal lung samples by immunohistochemistry (IHC). TGIF2 showed significantly higher expression in NSCLC samples than in normal tissues (Fig. 1a, b). Higher TGIF2 levels were observed in patients with NSCLC with higher pathological grades (Table 1). Compared with squamous cell carcinoma (*n* = 24) and large-cell undifferentiated carcinoma (*n* = 12), lung adenocarcinoma (*n* = 24) showed even higher TGIF2 expression (*P* < 0.001) (Fig. 1a, b).

**Table 1:** Correlation between TGIF2 expression and clinicopathological features in NSCLC.

Parameters	TGIF2 clinicopathological features in NSCLC			
		Low (9)	High (51)	*P* value
Sex	Female	2	13	0.835
	Male	7	38	
Age (years)	>55	6	33	0.909
	<55	3	18	
Tumor size	T1	3	1	0.001
	T2	6	37	
	T3–T4	0	13	
LN* metastasis	N0	9	16	<0.001
	N1	0	13	
	N2-N3	0	9	
Vessel invasion	M0	9	49	0.546
	M1	0	2	
Stage	I	9	8	<0.001
	II	0	16	
	III–IV	0	14	

NOTE: Statistical data on TGIF2 expression in relation to clinicopathological features for NSCLC microarray. *P*-values were calculated using the chi-square test

*LN: lymph node

We next explored the mRNA levels of TGIF2 in two cohorts from the TCGA database containing 515 human LUAD samples and 59 normal samples and a LUAD dataset from Gene Expression Omnibus (i.e., GSE32863) containing 36 LUAD samples and paired adjacent normal lung samples. Consistently, LUAD showed significantly higher expression of TGIF2 (Fig. 1c, d). Furthermore, increased expression of TGIF2 was also observed in the LUAD cell lines A549 and H1299 when compared with the normal lung cell line BEAS-2B (Fig. 1e). We also analyzed the correlation between the level of TGIF2 and the relapse-free survival (RFS) of patients with lung cancer in previously generated microarray data sets from 2437 patients with LUAD. We found that high TGIF2 expression was associated with poor clinical outcome in patients with LUAD (*P* < 0.001, Fig. 1f). Taken together, these results suggest a role for TGIF2 in promoting the progression of LUAD.

### TGIF2 enhances the CSC-like characteristics of LUAD cells and promotes metastasis

**Fig. 2:**
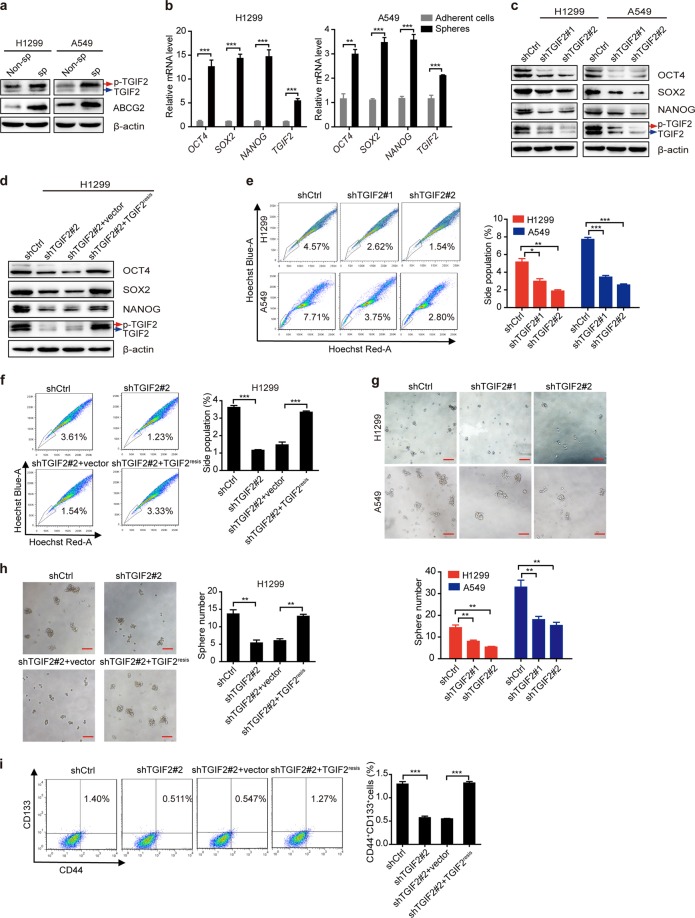
TGIF2 promotes the CSC-like characteristics of LUAD cells. **a** Western blot analysis of TGIF2, p-TGIF2, and ABCG2 expression in non-side populations (non-sp) and side populations (sp) of H1299 and A549 cells. **b** qRT-PCR analysis of *OCT4*, *SOX2*, *NANOG*, and *TGIF2* mRNA expression in adherent cells or spheres of H1299 and A549 cells. Data are shown as means ± SD. **P < 0.01, ***P < 0.001. **c** Western blot analysis of OCT4, SOX2, NANOG, TGIF2, and p-TGIF2 in TGIF2-silenced stable H1299 and A549 cells. **d** Western blots for OCT4, SOX2, NANOG, TGIF2, and p-TGIF2 in the indicated stable H1299 cells. **e** Flow cytometry analysis of side populations in TGIF2-silenced H1299 and A549 cells. Data are shown as means ± SD. *P < 0.05, **P < 0.01, ***P < 0.001. (**f**) Flow cytometry analysis of side populations in the indicated stable H1299 cell strains. Data are shown as means ± SD. ***P < 0.001. **g** Sphere formation ability of TGIF2-silenced H1299 and A549 cells. Scale bars: 50 μm. Data are shown as means ± SD. **P < 0.01. **h** Sphere formation ability of the indicated stable H1299 cell strains. Scale bars: 50 μm. Data are shown as means ± SD. **P < 0.001. **i** Flow cytometry analysis of CD133^+^ CD44^+^ double-positive cells in the indicated H1299-derived stable cell strains. Data are shown as means ± SD. ***P < 0.01.

As a member of the TALE proteins, which play crucial roles in many development processes,^24^ TGIF2 was reported to be able to reprogram liver cells to pancreas progenitor cells,^14^ suggesting the role of TGIF2 in regulating stemness and differentiation. To test whether TGIF2 regulates the stemness of LUAD cancer cells, we first examined the expression of TGIF2 in CSC-like cells. In CSC-like side populations of A549 and H1299 separated by FACS, we observed elevated expression of TGIF2 (Fig. 2a). Consistently, the CSC-like cells that could form spheres also showed increased expression of TGIF2 (Fig. 2b). To further explore whether TGIF2 could drive the stemness properties in LUAD cancer cells, we established TGIF2-silenced stable H1299 and A549 cells (Fig. S1a). Silencing TGIF2 significantly reduced the expression of OCT4, SOX2, and NANOG, key transcriptional factors driving the stemness properties,^25^ in H1299 and A549 cells (Fig. 2c), which could be rescued by the ectopic expression of shRNA-resistant TGIF2 (Fig. S1b and Fig. 2d). We also observed decreased side populations of TGIF2-silenced H1299 and A549 cells, which represented CSC-like cell populations (Fig. 2e), and once TGIF2 expression was rescued, the side populations were increased significantly (Fig. 2f). Silencing TGIF2 in H1299 and A549 cells could also dramatically weaken their sphere formation capacities (Fig. 2g), which could also be rescued by ectopically expressed TGIF2 (Fig. 2h). LUAD CSC-like cells were reported to highly express CD133 and CD44.^26,27^ We found that knocking down TGIF2 significantly reduced the CD133^+^ CD44^+^ subpopulation, which could be rescued by ectopically expressed TGIF2 as well (Fig. 2i).

**Fig. 3:**
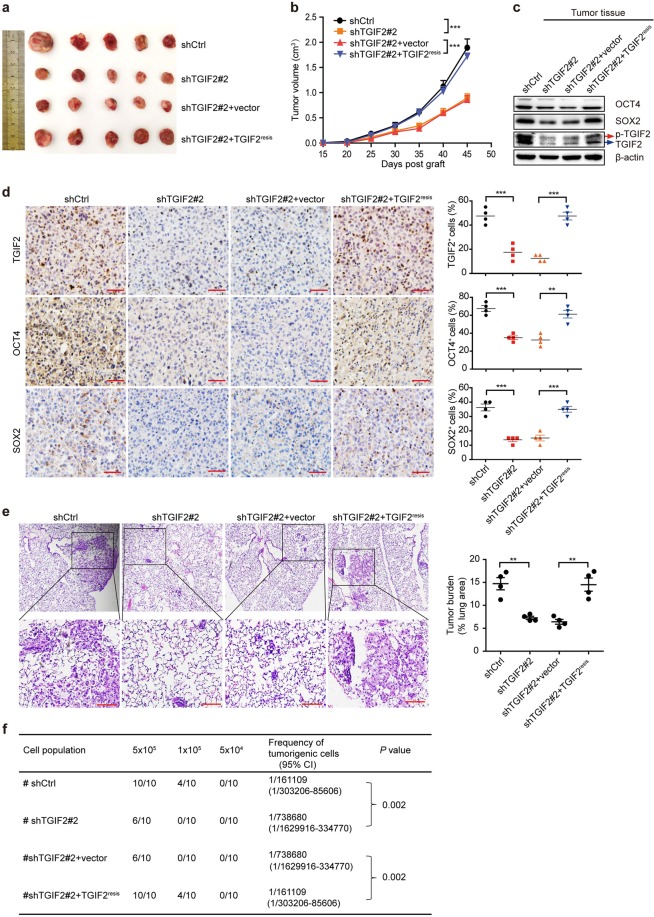
TGIF2 enhances the CSC-like characteristics of LUAD and promotes metastasis in vivo. **a** Tumor xenografts formed by subcutaneous inoculation of the indicated H1299 cell strains were dissected and the images were captured. **b** Tumor volumes were measured at the indicated time points. **c** The expression of OCT4, SOX2, and TGIF2 in tumor tissue lysates was examined by western blot. **d** IHC staining of TGIF2, OCT4, and SOX2 in the indicated xenograft tumor sections (scale bars: 50 μm, left panel) and quantification (*n* = 4, right panel). Data are shown as means ± SD. **P < 0.01, ***P < 0.001. **e** H&E staining was used to analyze lung metastasis from the indicated mice (scale bars: 250 μm, left panel), and the metastases were quantified (*n* = 4, right panel). Data are shown as means ± SD. **P < 0.01. **f** Tumor presence and tumorigenic cell frequency in tumors formed by the indicated H1299 stable cell strains were analyzed with a limiting dilution assay (http://bioinf.wehi.edu.au/software/elda/). Subcutaneous tumors were harvested on the 28^th^-day post grafting (*n* = 10). CI, confidence interval.

To gain insights into the importance of TGIF2 in promoting the stemness of LUAD cells in vivo, we performed xenograft experiments using H1299 cells. Silencing TGIF2 significantly reduced tumor growth, which could be rescued by the ectopic expression of shRNA-resistant TGIF2 (Fig. 3a, b). Furthermore, decreased levels of OCT4 and SOX2 were observed in TGIF2-silenced tumor cells, which could also be rescued by ectopically expressed TGIF2 (Fig. 3c, d). Furthermore, we observed that less TGIF2-silenced H1299 cells metastasized to the lung and initiated secondary tumors, which was reversed by ectopically expressed TGIF2 (Fig. 3e). In addition, a limited dilution xenograft assay revealed that 10^5^ of TGIF2-silenced H1299 cells xenografted in NOD/SCID mice showed a dramatically decreased incidence of initiating a tumor when compared with that of control cells (Fig. 3f), which could also be restored by increasing TGIF2 levels (Fig. 3f). Taken together, these results suggest a role for TGIF2 in maintaining the CSC-like characteristics of LUAD cells and promoting metastasis in vivo.

### TGIF2 promotes the CSC-like characteristics of LUAD cells by enhancing *OCT4* transcription

**Fig. 4:**
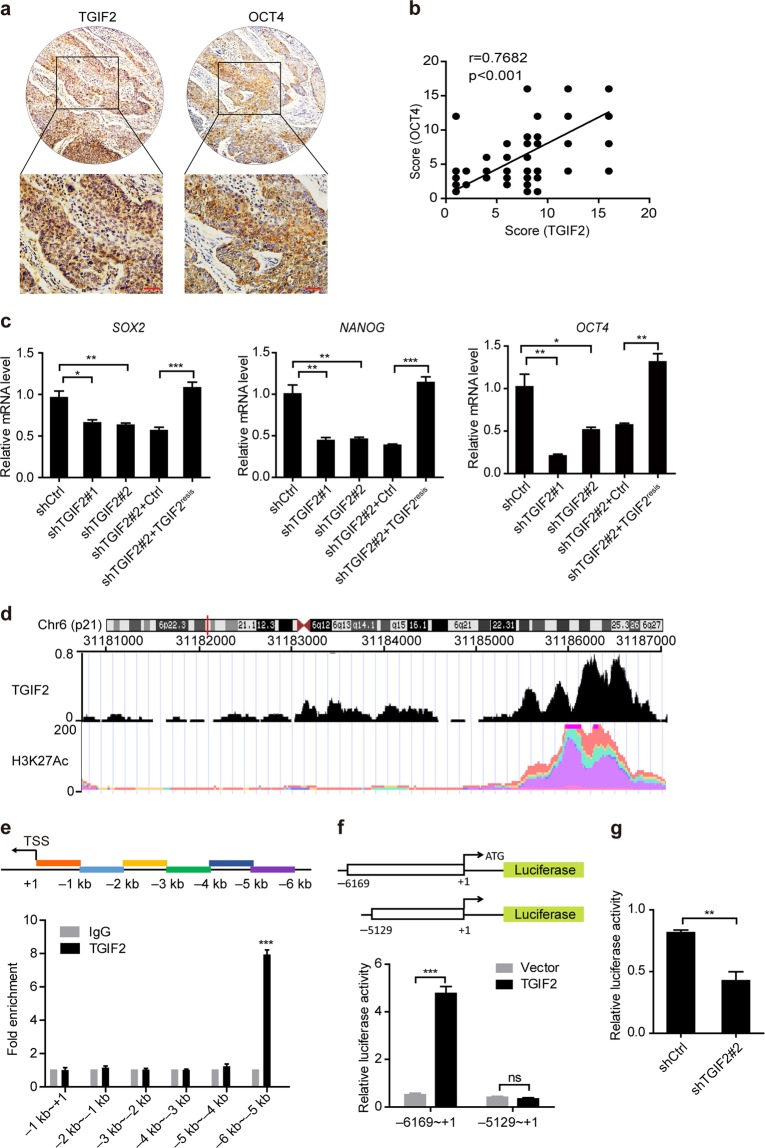
TGIF2 promotes the CSC-like characteristics of LUAD cells by enhancing *OCT4* transcription. **a** IHC staining of TGIF2 and OCT4 on the NSCLC tissue array. Scale bars: 50 μm. **b** Spearman’s correlation analysis of the expression of OCT4 and TGIF2 in human NSCLC tissues. **c** The mRNA levels of *SOX2*, *NANOG*, and *OCT4* were analyzed by qRT-PCR in the indicated cells. Data are shown as means ± SD. *P < 0.05, **P < 0.01, ***P < 0.001. **d** ChIP-Seq data from the Cistrome Data Browser database to show the TGIF2 binding sites and H3K27Ac on the human *OCT4* genomic locus. **e** The promoter region of the *OCT4* gene scanned by 6 amplicons is shown on the top. TGIF2 ChIP-qPCR of the *OCT4* promoter at the indicated regions in H1299 cells. Mouse IgG served as a negative control. Data are shown as means ± SD. ***P < 0.001. **f** Dual-luciferase assay to identify TGIF2-binding sites in the *OCT4* promoter in H1299 cells transfected with plasmids encoding the indicated sequences. Data are shown as means ± SD. ***P < 0.001. **g** Dual-luciferase assay to show *OCT4* promoter activity. The activity of the human *OCT4* promoter was examined by cotransfection of the luciferase reporter construct containing the region from −6169 to +1 of the *OCT4* promoter with shCtrl or shTGIF2#2 in H1299 cells. Data are shown as means ± SD. **P < 0.01.

In 60 human NSCLC samples, we found a good positive correlation between the levels of TGIF2 and OCT4 as determined by IHC (Fig. 4a, b). Additionally, silencing TGIF2 not only significantly reduced the protein levels of OCT4, SOX2, and NANOG in H1299 cells (Fig. 2c) but also decreased the mRNA levels of these genes (Fig. 4c), indicating that TGIF2 might promote the CSC-like characteristics of LUAD by regulating the transcription of these genes. To understand how TGIF2 regulated the transcription of these genes, we first searched the Gene Transcription Regulation Database (GTRD), which is a collection of many chromatin immunoprecipitation and deep DNA sequencing (ChIP-Seq) results, for TGIF2 binding sites on these genes. We only found that TGIF2 might bind to the –6096 ~ –4632 region of the *OCT4* promoter, where a high level of H3K27Ac was shown by the Cistrome Data Browser database, suggesting active transcription in this region (Fig. 4d). To further confirm these results, we performed a ChIP-qPCR assay to scan the human *OCT4* gene from −6 kb to the transcription start site (+1) for TGIF2 binding sites (Fig. 4e). Consistently, the TGIF2 binding site was found within the region from −6 kb to −5 kb of the *OCT4* promoter (Fig. 4e). TGIF2 was able to promote the −6 kb ~ +1 region-driven expression but not the −5 kb ~ +1 region-driven expression of the luciferase reporter gene (Fig. 4f), further suggesting that TGIF2 could enhance the transcription of *OCT4* by binding in the −6 kb to −5 kb region of its promoter. In addition, we found that silencing TGIF2 led to decreased reporter expression (Fig. 4g), confirming that TGIF2 acts as a positive regulator of the human *OCT4* promoter.

### TGIF1 does not compensate for the decreased TGIF2 in H1299 cells

Jonghwan Kim et al. demonstrated that mouse Tgif1 directly bound to the *Oct4* promoter to suppress Oct4 expression during the development of mouse embryos.^28^ To clarify the potential contribution of TGIF1, we overexpressed TGIF1 in the human lung cancer cell line H1299 and did not observe any changes in the expression of endogenous OCT4 (Fig. S2a) and the luciferase-reporter driven by the *OCT4* promoter (Fig. S2b), suggesting that in human lung cancer cells, TGIF1 was not able to regulate OCT4, which might be due to the low conservation between the mouse *Oct4* promoter and the human OCT4 promoter. We, therefore, aligned the sequence of the Tgif1-binding site (−1459 to +585) in the mouse *Oct4* promoter with that of the human *OCT4* promoter and found a very low similarity between these two promoters. These results revealed a specific role of TGIF2 in regulating human OCT4.

Additionally, we found that silencing TGIF2 caused decreased expression of TGIF1 (Fig. S2c, d), which ruled out the possibility that decreased OCT4 expression was due to compensation from the transcriptional repressor TGIF1.

### TGIF2 promotes LUAD progression in an EGFR signaling-dependent manner

**Fig. 5:**
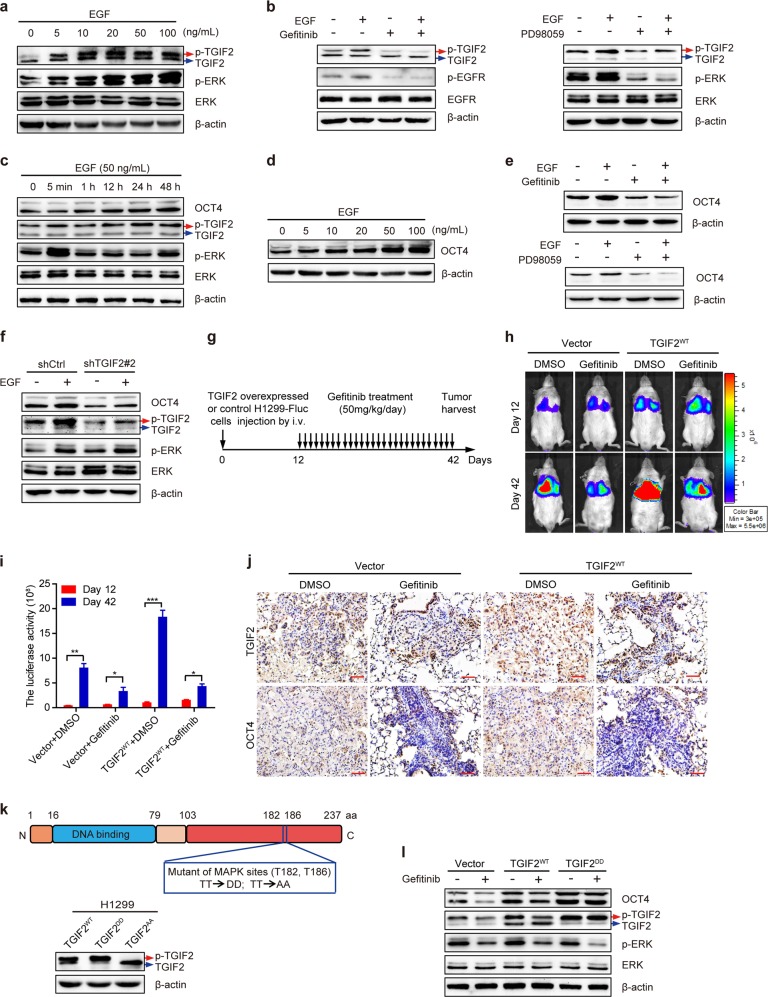
TGIF2 functions downstream of EGFR/RAS/ERK signaling to promote LUAD progression. **a** Western blot analysis showing the phosphorylation of TGIF2 in H1299 cells treated with the indicated dose of EGF for 5 min. **b** Western blot analysis of EGF-induced TGIF2 phosphorylation in H1299 cells in the presence of gefitinib (left panel) or PD98059 (right panel). **c** Western blots showing the time course of EGF-induced TGIF2 phosphorylation and OCT4 levels in H1299 cells. **d** The expression of OCT4 was analyzed by western blot in H1299 cells treated with different doses of EGF for 48 h. **e** Western blot analysis of OCT4 expression in H1299 cells stimulated with EGF in the presence or absence of gefitinib or PD98059. **f** Western blot analysis of OCT4 expression in TGIF2 knockdown and control H1299 cells in the presence or absence of EGF. **g** Schematic procedure of the in vivo mouse xenograft experiment. Mice were intravenously injected with H1299-firefly luciferase (H1299-Fluc) cells overexpressing TGIF2^WT^ or empty vector as a control. Twelve days later, the mice were orally administered 50 mg gefitinib/kg daily for 30 days. **h**, **i** Bioluminescence imaging of pulmonary metastasis at the indicated days (**h**), and the tumor sizes were quantified by bioluminescence intensity (**i**). Data are presented as means ± SD (*n* = 4, *P < 0.05, **P < 0.01, ***P < 0.001). **j** TGIF2 and OCT4 expression in lung tumors was detected by IHC staining. Scale bars: 50 μm. **k** Domain structure of TGIF2 to show the positions of the two MAPK sites (upper panel) and western blots for TGIF2 and p-TGIF2 in H1299 cells overexpressing TGIF2^WT^, TGIF2^DD^, or TGIF2^AA^ (lower panel). **l** Western blot analysis of OCT4, TGIF2, p-TGIF2, p-ERK and ERK expression in the indicated stable H1299 cells in the presence or absence of gefitinib.

Aberrant activation of EGFR drives tumorigenesis of various tumor types including lung carcinoma by activating downstream RAS-ERK and other pathways.^29^ TGIF2 is phosphorylated by the RAS-ERK pathway.^10^ We first confirmed that EGF was able to induce TGIF2 phosphorylation in H1299 cells (Fig. 5a), which could be inhibited by the EGFR inhibitor gefitinib and the MEK inhibitor PD98095 (Fig. 5b), suggesting that EGFR–RAS–ERK signaling could induce TGIF2 phosphorylation in LUAD cells as well. Consistently, we found that the expression of OCT4, as a target gene of TGIF2, was enhanced by EGF (Fig. 5c, d), which could also be inhibited by either EGFR or MEK inhibitors (Fig. 5e and Fig. S3a). Moreover, we detected the effect of EGF on the expression of OCT4 in TGIF2-silenced cells. Our results showed that TGIF2 silencing decreased the EGF-induced expression of OCT4 (Fig. 5f).

Next, we examined whether the role of TGIF2 in promoting LUAD progression was controlled by EGF/EGFR signaling in the H1299 xenograft mouse model (Fig. 5g). Inhibition of EGF/EGFR signaling by the widely utilized anti-tumor drug gefitinib greatly inhibited TGIF2^WT^-promoted metastasis of H1299 xenografts as monitored in vivo by the luciferase assay (Fig. 5h, i), which was further confirmed by counting the tumor burden shown by HE staining (Fig. S3b, c). TGIF2-induced OCT4 expression was also inhibited by gefitinib in LUAD cells in xenograft tumors (Fig. 5j and Fig. S3d).

To further investigate the important role of TGIF2 in the downstream of EGF/EGFR signaling, we generated a phosphorylation-mimicry TGIF2 mutant (i.e., T182D and T186D, or TGIF2^DD^ for short, Fig. 5k). In TGIF2^DD^-transfected H1299 cells, gefitinib failed to decrease OCT4 expression when compared with that of control cells (Fig. 5l), strongly suggesting that the effect of gefitinib on the CSC-like properties of LUAD cells is TGIF2-dependent.

### EGF/EGFR–RAS–ERK signaling increases the stability of TGIF2 to promote the CSC-like characteristics of LUAD cells

**Fig. 6:**
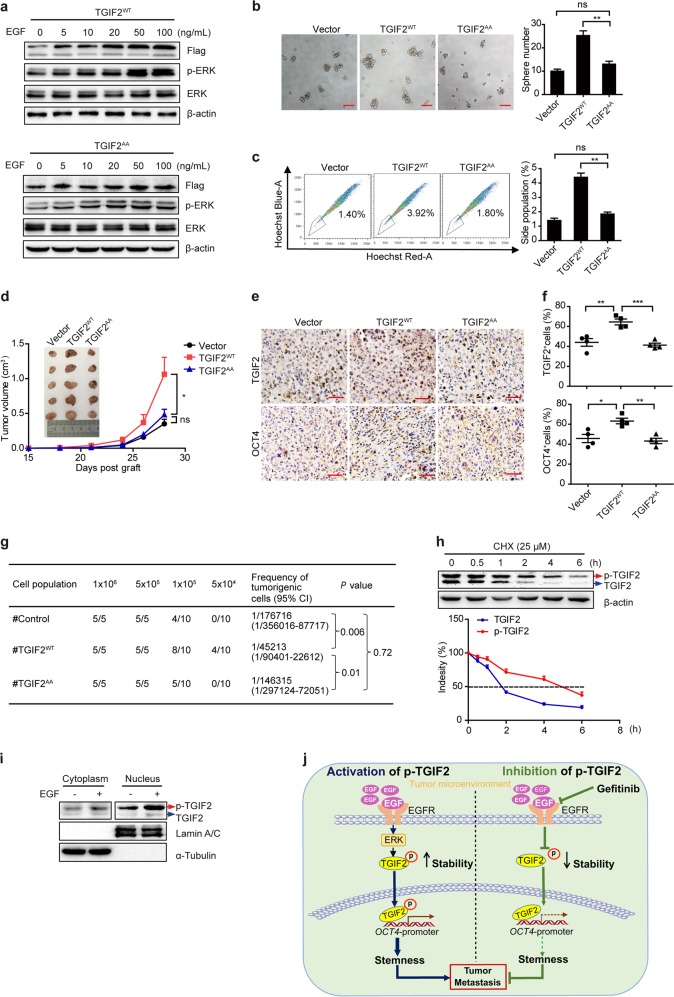
TGIF2 phosphorylation increases its stability to promote the CSC-like characteristics of LUAD cells. **a** Western blots for Flag-tagged TGIF2^WT^ and TGIF2^AA^ in H1299 cells treated with EGF for 5 min. **b** Sphere formation ability of TGIF2^WT^- and TGIF2^AA^-overexpressing H1299 cells. Scale bars: 50 μm. Data are shown as means ± SD. **P < 0.01. **c** Flow cytometry analysis of side populations in H1299 cells with ectopic expression of TGIF2^WT^ or TGIF2^AA^. Data are shown as means ± SD. **P < 0.01. **d** Subcutaneously inoculated tumor xenografts formed by TGIF2^WT^- or TGIF2^AA^ -overexpressing H1299 cells were harvested on the 28th-day post grafting, and tumor volumes were determined on the indicated days. *n* = 5 mice for each group. **e**, **f** TGIF2 and OCT4 in the indicated tumor xenografts were stained by IHC (**e**, Scale bars: 50 μm), and positively stained cells were quantified (**f**). Data are shown as means ± SD. * *P* < 0.05, **P < 0.01, ***P < 0.001. **g** Tumor presence and tumorigenic cell frequency in TGIF2^WT^-, TGIF2^AA^-, or vector-transfected tumor cells were analyzed with a limiting dilution assay. **h** Western blot analysis of TGIF2 levels in H1299 cells in the presence of 10 μM cycloheximide (CHX) for different periods (upper panel). TGIF2 levels were quantified and normalized to that of β-actin at different time points to calculate the half-lives of unphosphorylated and phosphorylated TGIF2 (lower panel). **i** The sub-cellular localization of unphosphorylated and phosphorylated TGIF2 in H1299 cells treated with or without 50 ng/mL EGF for 5 min was examined by cell fractionation and western blot. **j** Proposed model illustrating the role of TGIF2 downstream EGF/EGFR signaling in promoting LUAD stemness and metastasis. Activated EGFR by EGF phosphorylates TGIF2 via activation of RAS/ERK signaling to enhance the stability of TGIF2, which enhances *OCT4* transcription, resulting in increased cancer stemness and metastasis. However, inactive mutation or pharmacological inhibition (gefitinib) of phosphorylated TGIF2 decreases TGIF2 stability and eliminates the increased stemness by p-TGIF2.

To further understand whether phosphorylation of TGIF2 by EGFR–RAS–ERK is important for EGFR promoted stemness of cancer cells, we next compared the functions of wild type-TGIF2 (TGIF2^WT^) and phosphorylation-deficient TGIF2 mutants (i.e., T182A and T186A, or TGIF2^AA^ for short, Fig. 5k) in promoting the stemness of LUAD cells. First, we found that EGF could no longer induce the phosphorylation of TGIF2^AA^ compared to that of TGIF2^WT^ (Fig. 6a). As Fig. 6b shows, ectopic expression of TGIF2^WT^ promoted sphere formation in H1299 cells, while TGIF2^AA^ did not. Consistently, TGIF2^AA^ did not increase the side population of H1299 cells when compared with that of TGIF2^WT^ (Fig. 6c). In the xenograft mouse LUAD model, ectopic expression of TGIF2^AA^ did not promote tumor growth as TGIF2^WT^ did when compared with empty vector transfected cells (Fig. 6d). Furthermore, TGIF2^AA^-overexpressing tumor tissues showed no increased OCT4 levels while TGIF2^WT^-overexpressing tumors did (Fig. 6e, f). In addition, a limited dilution xenograft assay showed that tumor cells transfected with TGIF2^WT^ exhibited significantly increased tumor-initiating capacity in NOD/SCID mice compared with empty vector- or TGIF2^AA^- transfected cells (Fig. 6g). Thus, our results showed that phosphorylation of TGIF2 was necessary for promoting the CSC-like characteristics of LUAD cells.

Next, we explored how the phosphorylation of TGIF2 promoted the stemness of LUAD cells. TGIF has been reported to have a very short half-life.^30^ We examined the half-life of TGIF2 in H1299 cells and confirmed its short half-life of ~2 h (Fig. 6h). Interestingly, in comparison, phosphorylated TGIF2 showed a much longer half-life of 5 h (Fig. 6h). EGF could induce more phosphorylated TGIF2 accumulation in the nucleus of H1299 cells (Fig. 6i), which promoted the expression of OCT4.

In summary, our study demonstrated a critical role of TGIF2 in mediating EGF/EGFR–RAS–ERK signaling-induced stemness of LUAD cells, which could be a useful target to prevent LUAD.

## Discussion

In this study, we found that TGIF2 phosphorylation triggered by EGFR/RAS/ERK signaling enhanced the stability of TGIF2 to promote *OCT4* transcription, resulting in increased stemness and metastasis of LUAD cells (Fig. 6j).

TGIF2 has been identified as a transcription repressor recruiting HDACs to repress the activation of TGF-β-responsive transcription.^8^ Our study revealed that TGIF2 promoted *OCT4* transcription in an HDAC-independent manner. Importantly, TGIF2 phosphorylation as a consequence of EGFR/RAS/ERK signaling activation improved its stability and enhanced the CSC properties of LUAD. The phosphorylation-deficient TGIF2 mutant lost the activity to promote OCT4 expression and tumorigenesis in H1299 cells. The importance of OCT4 in tumorigenesis and as a prognostic marker has been previously reported.^31^ OCT4 maintains the CSC-like properties in lung cancer-derived CD133^+^ cells.^32^ Moreover, OCT4 is significantly associated with a poor prognosis of lung cancer.^33^ In the present study, the expression of TGIF2 and OCT4 was correlated in patients with NSCLC, corroborating that TGIF2 promotes LUAD stemness by upregulating OCT4 expression.

Additionally, TGIF2 is often involved in cancer by acting as a target of microRNAs,^34–38^ and few studies have investigated the mechanism of TGIF2 in cancer. In colon cancer, TGIF2 was reported to interact with PKM2 to recruit HDAC3 to the E-cadherin promoter, leading to EMT upon EGF stimulation. In LUAD, we showed here that EGF-induced phosphorylation of TGIF2 promoted *OCT4* transcription and enhanced the stemness of cancer cells. Whether TGIF2 in LUAD can suppress E-cadherin and induce the EMT process upon EGF stimulation remains elusive. Based on our findings, we suggest that TGIF2 can enhance the transcription of *OCT4* to regulate cancer stemness. Importantly, TGIF2 may cooperate with other cofactors to exert its transcriptional function in LUAD, which is consistent with other TALE homeoproteins showing a context-dependent action.^39^

In lung adenocarcinoma, EGFR mutation is one of the “driver” mutations, leading to constant activation of the PI3K/AKT and RAS/MEK/ERK pathways.^40^ In the present study, we found that TGIF2 phosphorylation triggered by EGFR/RAS/ERK signaling plays an important role in LUAD progression, which indicated that p-TGIF2 acts as an EGFR/ERK signaling-responsive factor to drive lung cancer development. Furthermore, constant activation of EGFR/ERK signaling caused by EGFR mutation may be one reason for the higher level of TGIF2 in LUAD patients. Gefitinib, as one of the anticancer therapeutics directed against EGFR, can inhibit tumor growth.^41^ Here, we found that gefitinib treatment inhibited tumor metastasis and decreased TGIF2 phosphorylation in vitro and in vivo. Moreover, gefitinib failed to decrease OCT4 expression in TGIF2^DD^-transfected H1299 cells when compared with expression in control cells, strongly suggesting that the effect of gefitinib on the CSC-like properties of LUAD cells is TGIF2-dependent. However, drug resistance invariably emerges in clinical therapy,^42^ which implies that other target agents should be imminently developed. Our findings indicated that targeting TGIF2 phosphorylation may be a potential strategy for LUAD therapy.

In summary, we demonstrated that TGIF2 was highly expressed in LUAD and upregulated OCT4 expression downstream of EGFR/RAS/ERK signaling, promoting cancer cell stemness and metastasis of LUAD cells. Our findings suggested that phosphorylation is necessary for TGIF2 to sustain the CSC characteristics and promote metastasis of LUAD cells, and that blocking TGIF2 phosphorylation might be an effective strategy for LUAD therapy.

## Materials and methods

### Cell culture

A549, H1299, and H460 human lung cancer cells were maintained in RIPM-1640 medium containing 10% fetal bovine serum (FBS), 100 U/mL penicillin and 0.1 mg/mL streptomycin. BEAS-2B cells were maintained in bronchial epithelial growth medium (BEGM) (Solarbio, Beijing, China) with growth factors, cytokines and supplements (BR, Lonza, Switzerland) in the SingleQuot® supplement kit. Cells were maintained at 37 °C in a humidified atmosphere with 5% CO_2_. All cell lines were recently authenticated by cellular morphology and short tandem repeat analysis at Microread Inc. (Beijing, China).

For the activation of EGFR/ERK signaling, H1299 cells were starved overnight and incubated with human recombinant EGF (Invitrogen, Carlsbad, CA, USA) for 0–48 h or treated with 0–100 ng/mL EGF for 5 min or 48 h. For inhibition of EGFR/ERK signaling, cells were pre-incubated with 10 μM gefitinib (MedChem Express, New Jersey, USA) or 20 μM PD98059 (MedChem Express, New Jersey, USA) for 1 h and then exposed to 50 ng/ml EGF for 5 min or 48 h.

### Vector construction and stable cell strain establishment

To stably knock down TGIF2 in LUAD cells, we inserted control or TGIF2-targeting shRNA templates into the pLV-H1-EF1a-Puro vector (Biosettia, San Diego, CA, USA) (named shCtrl, shTGIF2#1 and shTGIF2#2). The sequences are presented in Supplementary Table S1. For the stable overexpression of TGIF2 or TGIF2 mutant and TGIF1 in mammalian cells, the human full-length TGIF2^WT^ gene, phosphorylation-deficient TGIF2^AA^ mutant (i.e., T182A and T186A) gene, phosphorylation-mimicry TGIF2^DD^ mutant (i.e., T182D and T186D) gene, and human full-length TGIF1 gene were cloned into the pLV-EF1a-MCS-IRES-Bsd vector (Biosettia, San Diego, CA, USA).^43^ To rescue TGIF2 expression in TGIF2-silenced cells, shTGIF2#2-resistant TGIF2 was prepared and sub-cloned into the pLV-EF1a-MCS-IRES-Bsd vector between the BamH I and Mlu I sites. The primers for the modification of TGIF2 cDNA are described in Supplementary Table S2.

To establish stable cell strains, A549 and H1299 cells were infected with lentivirus expressing shTGIF2, followed by clone selection using 2 μg/mL puromycin to establish TGIF2-silenced stable H1299 and A549 cells. TGIF2-silenced stable H1299 (shTGIF2#2) cells were infected with lentivirus carrying pLV-EF1a-Flag-TGIF2^resis^-IRES-Bsd followed by selection with 10 μg/mL blasticidin to establish shRNA-resistant TGIF2 (shTGIF2#2 + TGIF2^resis^) cells. H1299 cells were infected with lentivirus carrying pLV-EF1α-Flag-TGIF2^WT^-IRES-Bsd or pLV-EF1α-Flag-TGIF2^DD^-IRES-Bsd or pLV-EF1α-Flag-TGIF2^AA^-IRES-Bsd plasmids to generate TGIF2^WT^-, TGIF2^DD^- and TGIF2^AA^-overexpressing cells.^44^

### Quantitative RT-PCR (qPCR)

qRT-PCR was performed following a previous protocol.^45^ The primers used in this assay are listed in Supplementary Table S3.

### Western blot analysis

Protein expression detected by western blot was carried out according to the established protocols described previously.^46^ The primary antibodies used in this assay are listed in Supplementary Table S4. All western blot results are provided as representative images from three independent experiments.

### Nuclear fractionation analysis

Cells were harvested, and the cytoplasmic and nuclear fractions were separated and extracted with an NE-PER Nuclear and Cytoplasmic Extraction Kit (Thermo Fisher Scientific Inc. MA, USA). TGIF2 proteins were detected by western blot.^47^

### Immunohistochemistry (IHC)

The tissue microarrays used for analysis of the expression of TGIF2 and OCT4 in NSCLCs were purchased from Alenabio Inc. (cat. # LC2081, Shanxi, China). The patients’ clinicopathological information is shown in Supplementary Table S5. The expression levels were scored according to the established protocols described previously.^46^

### Immunofluorescence

Cells grown on glass slides or tumor tissue slices were fixed in 4% paraformaldehyde and labeled with primary antibodies overnight at 4 °C, followed by incubation with appropriate fluorescent secondary antibodies at room temperature for 1 h. Nuclei were stained with DAPI, and images were captured using a Leica DM4000 upright microscope or confocal fluorescence microscope (Nikon).

### Side population assay

Cells were suspended at a density of 1 × 10^6^ cells/mL and then incubated with 7 μg/mL (for A549) or 2 μg/mL (for H1299) Hoechst 33342 (Sigma-Aldrich, St. Louis, MO, USA) at 37 °C for 60 min. Verapamil (Sigma-Aldrich, St. Louis, MO, USA) served as a negative control. Samples were analyzed by flow cytometry (FACSCalibur, BD Biosciences, San Jose, CA, USA) and analyzed with FlowJo software (Tree Star, Inc., Ashland, OR, USA).

### Sphere formation assay

Cells were collected and rinsed to remove serum and then dissociated to single-cell suspension in serum-free RIPM-1640 medium supplemented with 20 ng/mL EGF, 20 ng/mL human recombinant basic fibroblast growth factor (bFGF) and 2% B27 supplement (Invitrogen, Carlsbad, CA, USA). Cells were subsequently cultured in ultra-low attachment 24-well plates at a density of 1000 cells per well. After 7 days (for A549 cells) or 9 days (for H1299 cells), the spheres (determined as >20 cells/spheroid) were counted.

### Flow cytometry

Tumor cells were stained with phycoerythrin-conjugated mouse anti-human CD133 (BD Biosciences) and fluorescein isothiocyanate-conjugated mouse anti-human CD44 (BD Biosciences) for flow cytometric analysis. Non-stained cells were included as a control. One microliter of antibody was added to 100 μL of cell suspension (1 × 10^6^ cells/mL), and the mixture was incubated at 4° C for 1 h. Data were acquired on a flow cytometer and analyzed with FlowJo software.

### Dual-luciferase assay

The luciferase activity was determined using the Dual-Luciferase Reporter assay system (Promega, Madison, WI, USA). The promoter regions (−6169 ∼ +1 and −5129 ∼ + 1) of the *OCT4* gene were amplified by PCR and cloned into the pGL3-basic vector to create the pGL3-OCT4 firefly luciferase reporter plasmids. The primers used in this assay are listed in Supplementary Table S2. For reporter assays, H1299 cells were transiently transfected with pGL3-OCT4 reporter plasmid and Flag-TGIF2^WT^ or empty vector as a control. Firefly luciferase activity was normalized to Renilla luciferase activity for all samples to yield relative luciferase activity.

### Chromatin immunoprecipitation (ChIP)

ChIP was carried out using the EZ-Zyme Chromatin Prep Kit (Millipore, Billerica, MA, USA) according to the manufacturer’s protocol. Anti-TGIF2 antibody was used to precipitate DNA cross-linked with TGIF2, and anti-mouse IgG was also used as a negative control. qPCR was performed to detect DNA fragments of the *OCT4* promoter region. The primers used are listed in Supplementary Table S6.

### Xenograft tumor model and drug administration in vivo

All the in vivo mouse experiments were approved by the Ethics Committee of Nankai University. NOD/SCID male mice at 6–8 weeks of age were allocated randomly to each group (*n* ≥ 4). Cells were subcutaneously injected into each mouse. Tumor size (mm^3^) was measured with calipers and calculated by the following formula: volume (mm^3^) = (width^2^ (mm^2^) × length (mm))/2. The individual measuring the tumor sizes was blinded to the treatments. Primary tumor tissues and lungs were formalin fixed, paraffin embedded, and sectioned for further analysis. Subcutaneous tumors formed by 1 × 10^6^ H1299 cells expressing shTGIF2#2, shTGIF2#2 + vector, shTGIF2#2 + TGIF2^resis^, or empty vector separately were dissected at 45 days after implantation.

For limited dilution transplantation, 1 × 10^6^, 5 × 10^5^, 1 × 10^5^ or 5 × 10^4^ cells were subcutaneously injected into NOD/SCID male mice, and 28 days after implantation, tumor tissues were harvested.

For gefitinib treatment, NOD/SCID mice were injected with 1 × 10^6^ H1299-firefly luciferase (H1299-Fluc) cells stably transfected with TGIF2^WT^ or empty vector via the tail vein. Twelve days after injection, the mice received 50 mg/kg gefitinib daily by intragastric administration for 30 days, and DMSO was used as a control. Tumor volumes were calculated, and bioluminescence images were captured.

### Patient datasets

Survival analyses were conducted with the online tool (http://kmplot.com/analysis/index.php?p=service&cancer=lung) using the 2015 version. Patients with lung adenocarcinoma (*n* = 2437) were selected for the overall survival assay. Log-rank was automatically computed.^48^ TGIF2 transcript levels in primary tumors and normal tissues in LUAD were analyzed at UALCAN (http://ualcan.path.uab.edu/index.html). The gene expression profiles of 36 pairs of lung adenocarcinoma (LUAD) and paracarcinoma tissues were downloaded from https://www.ncbi.nlm.nih.gov/gds (accession number: GSE32863).

### Statistical analysis

Kaplan–Meier survival curves were created using the log-rank test for TCGA data to compare the TGIF2 high group with the TGIF2 low group. All data were analyzed using GraphPad Prism5 software (GraphPad Software, San Diego, CA, USA). The results are expressed as means ± SD with the exception of human sample and animal model data, which are expressed as means ± SEM. *P-*values were calculated using a two-tailed Student’s *t*-test (two groups) or one-way ANOVA (more than 2 groups) unless otherwise noted. The results were considered statistically significant when *P* < 0.05.^49^

## Supplementary information

Supplementary information
