# Supplementary material for: TGIF2 promotes the progression of lung adenocarcinoma by bridging EGFR/RAS/ERK signaling to cancer cell stemness
Source: Signal Transduct Target Ther. 2019 Dec 13;4:60. doi: 10.1038/s41392-019-0098-x (PMC6908606; doi:10.1038/s41392-019-0098-x)
Supplement: Supplementary file 1 — Supplementary information [file 41392_2019_98_MOESM1_ESM.docx]

Supplementary Materials for

TGIF2 promotes the progression of lung adenocarcinoma by bridging EGFR/RAS/ERK signaling to cancer cell stemness

Renle Du,^1^ Wenzhi Shen,^2^ Yi Liu,^1^ Wenjuan Gao,^1^ Wei Zhou,^1^ Jun Li,^1^ Shuangtao Zhao,^1^ Chong Chen,^3^ Yanan Chen,^1, 5, 6^ Yanhua Liu,^1, 5, 6^ Peiqing Sun,^4^ Rong Xiang,^1, 5, 6^ Yi Shi,^1, 5, 6^***** and Yunping Luo^3^*****

^1^Department of Immunology, School of Medicine, Nankai University, Tianjin 300071, China

^2^Department of Pathology and Institute of Precision Medicine, Jining Medical University, Jining 272067, China

^3^Department of Immunology, Institute of Basic Medical Science, Chinese Academy of Medical Science, School of Basic Medicine, Peking Union Medical College, Beijing 100005, China

^4^Department of Cancer Biology, School of Medicine, Wake Forest University, Winston-Salem, NC 27157, USA.

^5^2011 Project Collaborative Innovation Center for Biotherapy of Ministry of Education, Tianjin 300071, China

^6^Tianjin Key Laboratory of Tumour Microenvironment and Neurovascular Regulation, Tianjin 300071, China

* Correspondence to: Yunping Luo, Tel: 86.010.69156475, E-mail: [ypluo@ibms.pumc.edu.cn](mailto:ypluo@ibms.pumc.edu.cn); Yi Shi, Tel: 86.022.23509482, E-mail: [yishi@nankai.edu.cn](mailto:yishi@nankai.edu.cn).

**This file includes:** Figures S1 to S3, Tables S1 to S6.

**
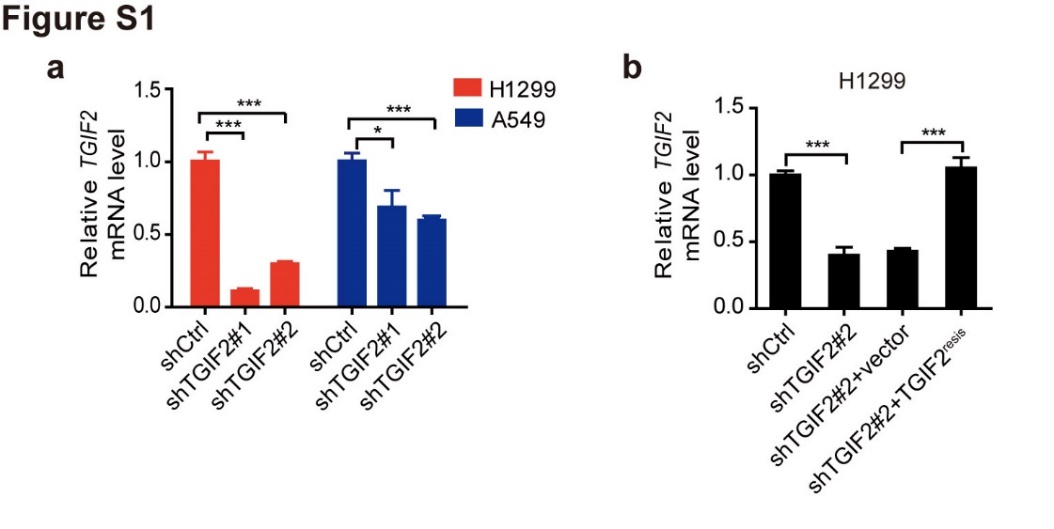
**

**Figure S1. The mRNA levels of *TGIF2* in TGIF2-silenced and TGIF2-rescued LUAD cells.**

(**a**) qRT-PCR analysis of *TGIF2* mRNA levels in H1299 and A549 cells stably transfected with two shRNAs targeting TGIF2 (shTGIF2#1 and shTGIF2#2). Data are presented as the means ± SD. **P*<0.05, ****P*<0.001.

(**b**) qRT-PCR analysis of *TGIF2* mRNA levels in H1299 cells stably transfected with shTGIF2#2 and shTGIF2#2-TGIF2^resis^. Data are presented as the means ± SD. ****P*<0.001.


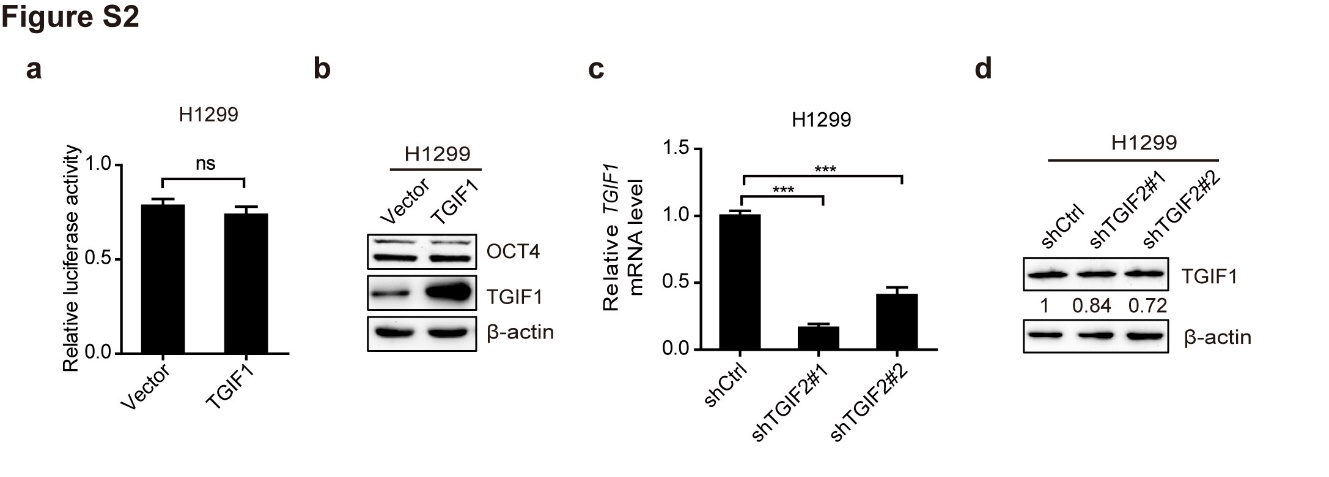


**Figure S2. TGIF1 does not compensate for the decreased TGIF2 in H1299 cells**

(**a**) Dual-luciferase assay to show the *OCT4* promoter activity. The activity of the human *OCT4* promoter was examined by co-transfection of the luciferase reporter construct containing the region from −6169 to +1 of the *OCT4* promoter with vector or TGIF1 in H1299 cells. Data are shown as the means ± SD. ***P*<0.01.

(**b**) Western blot analysis of OCT4 in TGIF1-overexpressing H1299 cells.

(**c**) qRT-PCR analysis of *TGIF1* mRNA levels in H1299 cells stably transfected with shTGIF2#1 or shTGIF2#2. Data are presented as the means ± SD. ****P*<0.001.

(**d**) Western blots for TGIF1 in indicated stable H1299 cells. The mean values were shown at the bottom of the blot.

**
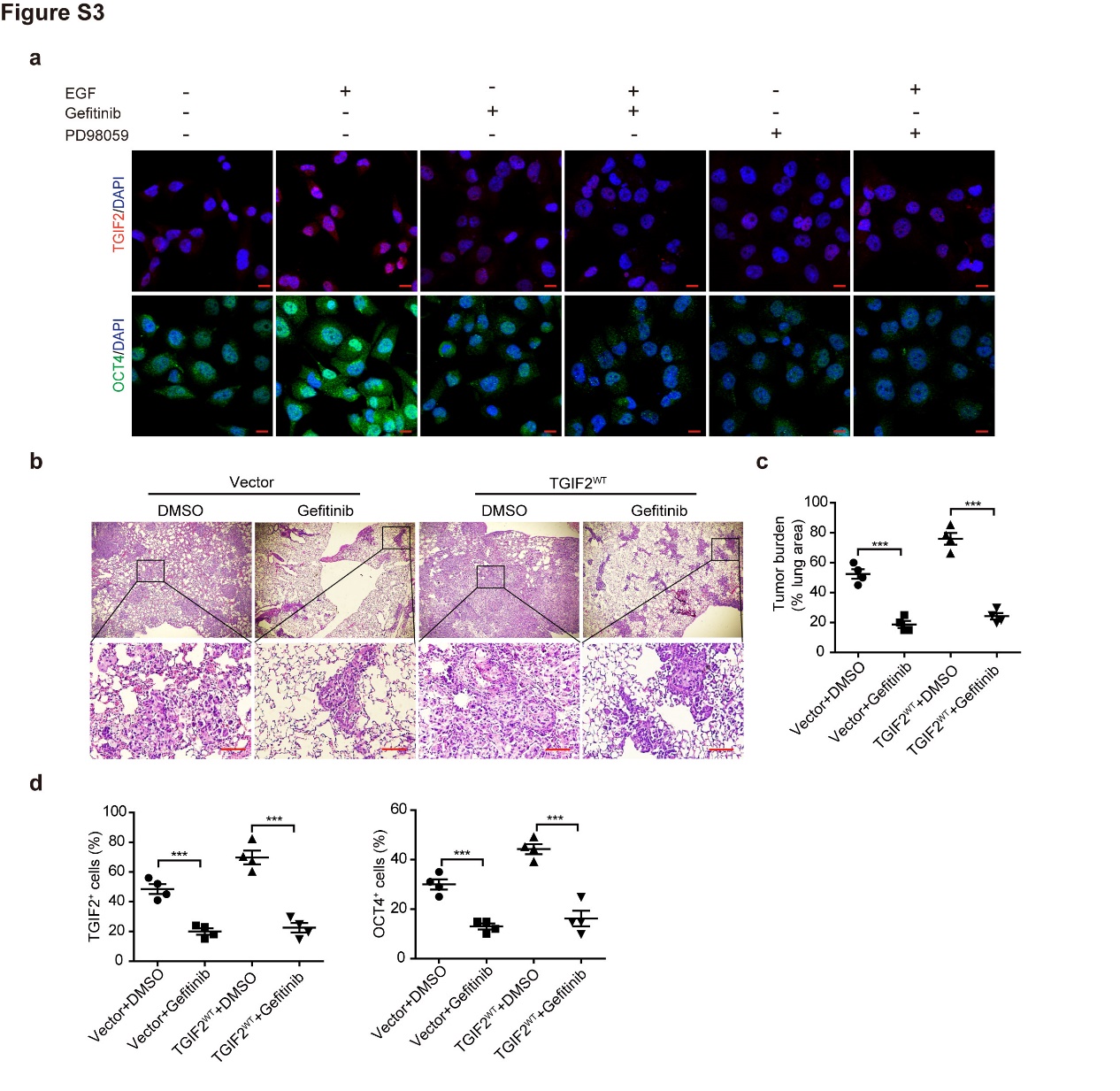
**

**Figure S3. TGIF2-induced OCT4 expression and lung metastasis were inhibited by gefitinib**

(**a**) EGF induced the accumulation of TGIF2 in the nucleus and promoted OCT4 expression in H1299 cells. H1299 cells were FBS-starved overnight, pre-incubated with 10 μM gefitinib or 20 μM PD98059 for 1 h, and then exposed to 50 ng/mL EGF for 5 min (for TGIF2) or 48 h (for OCT4) and then examined by immunofluorescence for TGIF2 and OCT4. Scale bars: 20 μm.

(**b, c**) The metastases in lungs of the indicated mice were shown by H&E staining (**b**, scale bars: 100 μm) and quantified (**c**). Data are presented as the means ± SD. *** *P*<0.001.

(**d**) The TGIF2 and OCT4 expression in lung tumors were quantified as positively stained cells. Data are shown as the means ± SD. ****P*<0.001.

**Supplementary Table S1. The template sequences of shRNAs targeting TGIF2 and negative control shRNA**

| **shRNA** | **Sequences (5’-3’)** |
| --- | --- |
| shTGIF2#1 | AAAAGCACACTCCCATCCCTTTAGTTTGGATCCAAACTAAAGGGATGGGAGTGTGC |
| shTGIF2#2 | AAAAGCACACTTACTCTGCTGACCATTGGATCCAATGGTCAGCAGAGTAAGTGTGC |
| shCtrl | AAAAGCTACACTATCGAGCAATTTTGGATCCAAAATTGCTCGATAGTGTAGC |

Supplementary Table S2. Primer sequences for cloning

| **Clone** | **Forward Sequences (5’-3’)** | **Reverse Sequences (5’-3’)** |
| --- | --- | --- |
| Flag-TGIF2^WT^ | CGGGATCCGCCACCATGGATTACAAGGATGACGACGATAAGGCCCGGATGTCGGACAGTGATCTAGGT | CGACGCGTCTACTGGGGATTTTCAGAGAC |
| TGIF2^AA^ | GGTGGACTCTTCAACGCGCCACCACCCGCACCCCCAGAGCAGG | CCTGCTCTGGGGGTGCGGGTGGTGGCGCGTTGAAGAGTCCACC |
| TGIF2^DD^ | GGTGGACTCTTCAACGACCCACCACCCGACCCCCCAGAGCAGG | CCTGCTCTGGGGGGTCGGGTGGTGGGTCGTTGAAGAGTCCACC |
| Modified Flag-TGIF2^WT^ sequences with mutation at shTGIF2#2 targeting sites | GTACTCTAACATTACTCACGA |  |
| TGIF1 | CGGGATCCATGGTTCTAGCGCAGAGCC | CGACGCGTTTAAGCTGTAAGTTTTGCCTGAAG |
| –6169~+1 | CGAGCTCCACCTTTTACATGAGCAGGTTTG | CGGCTAGCCAGGCCGGCGGAATCAC |
| –5129~+1 | CGAGCTCCGACTTAGATTGTGTGATACTTTGGT | CGGCTAGCCAGGCCGGCGGAATCAC |

Supplementary Table S3. Primer sequences for qRT-PCR

| **Gene** | **Forward Sequences (5’-3’)** | **Reverse Sequences (5’-3’)** |
| --- | --- | --- |
| *TGIF2* | CGGACAGTGATCTAGGTGAGGACG | GGGAAATGGTAAACTGATTAGGGTCTT |
| *SOX2* | GCCTGGGCGCCGAGTGGA | GGGCGAGCCGTTCATGTAGGTCTG |
| *OCT4* | GCTCGAGAAGGATGTGGTCC | CGTTGTGCATAGTCGCTGCT |
| *NANOG* | TCTGGACACTGGCTGAATCCT | CGCTGATTAGGCTCCAACCAT |
| *TGIF1* | CCCCAGTGCTCCTTTTCCAC | GAAGGTAGGCCAAAGTGCCG |

Supplementary Table S4. Antibodies used in the study

| **Antibody** | **Company (Art.No.)** | **Apply (dilution)** |
| --- | --- | --- |
| TGIF2 | Santa Cruz (sc-390870) | WB (1:500), ChIP (6μg for 10^6^ cells) |
|  | Abcam (ab155948) | IHC (1:100), IF(1:200) |
| β-actin | Santa Cruz (sc-47778) | WB (1:10000) |
| α-Tubulin | Cell signaling (2144) | WB (1:1000) |
| Lamin A/C | Cell signaling (4777) | WB (1:1000) |
| Flag | Sigma-Aldrich (F1840) | WB (1:1000) |
| ERK | ZSGB-BIO (ZS-94) | WB (1:1000) |
| p-ERK | ZSGB-BIO (ZS-7976) | WB (1:1000) |
| EGFR | Cell signaling (2232) | WB (1:1000) |
| p-EGFR | Cell signaling (4407) | WB (1:1000) |
| NANOG | Abcam (ab80892) | WB (1:1000) |
| OCT4 | Abcam (ab19857) | WB (1:1000),  IHC (1:100),  IF (1:200) |
| OCT4 | Proteintech (11263-1-AP) | WB (1:1000) |
| SOX2 | Abcam (ab92494) | IHC (1:100) |
|  | Santa Cruz (sc-20088) | WB (1:5000) |
| TGIF1 | Santa Cruz (sc-17800) | WB (1:500) |

Supplementary Table S5. Clinicopathological characteristics of patients from NSCLC tissue array

| **Characteristics** | **Patients (n=60)** |
| --- | --- |
| **Histologic type** |  |
| Large cell undifferentiated carcinoma | n=12 |
| Adenocarcinoma | n=24 |
| Squamous cell carcinoma | n=24 |
| **Grade** |  |
| 1 | n=16 |
| 2 | n=18 |
| 3 | n=23 |
| **Stage** |  |
| Ⅰ | n=17 |
| Ⅱ | n=16 |
| Ⅲ-IV | n=14 |
| **Tumor size** |  |
| T1 | n=4 |
| T2 | n=43 |
| T3-T4 | n=13 |
| **Lymph node metastasis** |  |
| N0 | n=25 |
| N1 | n=13 |
| N2 | n=9 |
| **Vessel invasion** |  |
| M0 | n=58 |
| M1 | n=2 |

**Supplementary Table S6. Primer sequences for ChIP-qPCR assay**

| **Fragments of *OCT4* promoter** | **Forward Sequences (5’-3’)** | **Reverse Sequences (5’-3’)** |
| --- | --- | --- |
| **–1222~+1** | AAATGGGCAGGAAGAGGT | CTTGGTCGCTGTGCAGTC |
| **–2196~–1177** | AGCCCAGGGAATAACCAA | AATAGCGGCACAAGCACC |
| **–3168~–2150** | CCCAGCCACCTTACTCCT | CTCCCATCCACGTACAGC |
| **–4172~–3147** | CCCTGATTGTCCCTCTTC | TTGGCATGACTTATTACTCC |
| **–5129~–4153** | GCATTAGAGGTGGGTTGG | TGCCTTGGCTTACAGGTT |
| **–6169~–5169** | CCCCATGTAATTCTTTCC | CTGCCTGTCTTTGGTTTT |
